# Selections and Abstracts

**Published:** 1902-08

**Authors:** 


### *SELECTIONS AND ABSTRACTS.*

---

### Studies in the Twelfth Census.

By H. E. Handerson, M.D., Ph.D., Cleveland.

That the study of census reports is not an exhilarating occupation is a proposition which will, I suspect, arouse no opposition from the members of the medical profession. Barring a table of logarithms, I know no work so well adapted to acquaint one with what Virchow has aptly called "the brute force of figures" as the official reports of the Director of the Census. Yet these reports are filled with interesting and important facts, the general knowledge of which is a great desideratum. The purpose of the following paper is to develop and emphasize certain facts, demonstrated indeed, in these reports, but liable to be either entirely overlooked, or at least undervalued in the wilderness of official figures.

Undoubtedly the chief cause of aversion to the ordinary census reports is the large numbers in which their results are tabulated. The computations of the average doctor may usually be made with two figures, exceptionally they may require three and, in the rarest of cases, even four. But a number requiring for its expression as much as seven or eight digits is to him a pure mental abstraction, incommensurable by any facts within his own experience and therefore purely speculative—*vox et prœterea nihil.* The statement *e. g.*, that the population of the United States in 1900 consisted of 38,816,448 males and 37,178,127 females conveys to the average mind merely the general idea that the male population slightly exceeded the female, but without suggesting even approximately the *rate* of inequality in the numbers of the two sexes. But suppose now we place this same statement in a different form : suppose we say that in the year 1900 *each thousand* of our population consisted of 511 males and 489 females, and we see at once, not only the excess of males, but likewise its relative proportion. Indeed, the sympathetic philanthropist may almost extend either his sympathy or his congratulations to the twenty-two males in each thousand, for whom, according to the figures, providence has provided (barring death or divorce) no female companions. Yet all that we have done has been simply to reduce the figures of the census to an easily commensurable decimal form, which at once unravels the intricate relations of the larger numbers, renders them easily understood and easily remembered, and even sheds a glimmer of personality upon the figures presented. This is of course nothing new. The system of percentages has been always employed in the preparation of census reports, and the system proposed is merely an extension of the former. The selection of one thousand, instead of one hundred, for the unit of comparison permits increased accuracy with the avoidance of fractions, and without the use of numbers too large to be readily appreciable.

We have all at least heard of the picture called "composite photographs," representations in which the characteristic features of a number of persons are so combined as to produce a picture of a class; a likeness in which the *common* characteristics of the various individuals are preserved, but mere individual peculiarities are eliminated. Something of the same kind may be accomplished with the statistics of a country, a State or even a city, with the decided advantage that such pictures give us a clear idea of the social and other characteristics of the community under investigation, and enable us to compare conveniently different communities.

The *modus operandi* is simple and easy. The community is divided into representative groups of one thousand individuals, which for the sake of conciseness we will call "chiliads." Each chiliad is credited with its proportionate share of the characteristics of the given community, and we thus finally attain a composite picture (what I have called a "chiliagraph"), which accurately represents the entire community and may be easily studied in itself or in comparison with the chiliagraphs of other communities.

Table I. below presents such a chiliagraph of the United States in 1900, exhibiting the characteristics of the various grand divisions* of our country in relation to sex, birth, color and age. To this is added a picture of the United States as a whole. It should be premised, however, that only the States and organized Territories of the United States are included in this table. Alaska, Hawaii, Porto Rico and other island possessions, whose social and political conditions are as yet too unsettled to furnish trustworthy data for study, have been, for this reason, excluded from consideration.

TABLE I.

CHILIAGRAPH OF THE UNITED STATES, 1900.

| Grand Divisions | N. Atlantic | S. Atlantic | N. Central | S. Central | Western | U. S. |
|---|---|---|---|---|---|---|
| Population in Chiliads | 21,047 | 10,443 | 26,333 | 14,080 | 4,091 | 75,995 |
| Increase 1890—1900 { Chiliads | 3,640 | 1,586 | 3,923 | 2,910 | 989 | 13,047 |
| Increase 1890—1900 { Per Cent | 20.9 | 18 | 17.5 | 26 | 31.9 | 20.70 |
| Land Area—Sq. Miles | 162,103 | 268 620 | 753,550 | 610,215 | 1,175,550 | 2,970,038 |
| Population to Sq. Mile | 129.8 | 38.8 | 34.9 | 23.07 | 3.5 | 25.6 |
| Sex { Females | 500 | 500 | 516 | 510 | 562 | 511 |
| Sex { Males | 500 | 500 | 484 | 490 | 438 | 489 |
| Birth { Native | 774 | 979 | 842 | 975 | 793 | 864 |
| Birth { Foreign | 226 | 21 | 158 | 25 | 207 | 136 |
| Color { White | 981 | 642 | 979 | 697 | 947 | 879 |
| Color { Negro | 18 | 357 | 19 | 298 | 7 | 116 |
| Color { Others | 1 | 1 | 2 | 5 | 46 | 5 |
| Approximate Age Scale. { 0–4 | 108 | 140 | 116 | 144 | 105 | 121 |
| Approximate Age Scale. { 5–20 | 300 | 383 | 340 | 393 | 305 | 343 |
| Approximate Age Scale. { 21 plus | 592 | 477 | 544 | 463 | 590 | 536 |

The first section of this table furnishes, as will be seen, the population of each grand division of our country, expressed in chiliads. The actual population (to within less than five hundred) may be determined by simply annexing three ciphers to the numbers of the table.

The second section gives us, in both chiliads and percentages, the increase of population in each division during the last decade. It will probably surprise most of us to learn that while the absolute increase of the North Central division, in which we are located

* For the convenience of those not entirely familiar with the divisions of the census reports, the following tabular view is added:

North Atlantic Division—Maine, New Hampshire, Vermont, Massachusetts, Rhode Island, Connecticut, New York, New Jersey, Pennsylvania.

South Atlantic Division—Delaware, Maryland, District of Columbia, Virginia, West Virginia, North Carolina, South Carolina, Georgia, Florida.

North Central Division—Ohio, Indiana, Illinois, Michigan, Wisconsin, Minnesota, Iowa, Missouri, North Dakota, South Dakota, Nebraska, Kansas.

South Central Division—Kentucky, Tennessee, Alabama, Mississippi, Louisiana, Arkansas, Indian Territory, Oklahoma, Texas.

Western Division—Montana, Idaho, Wyoming, Colorado, New Mexico, Arizona, Utah, Nevada, Washington, Oregon, California.

has been larger than that of any other division of the country, its *rate* of increase has been also the smallest. The largest rate of increase (excluding the Western division, still largely in process of settlement) is shown by the South Central division, a section which we are not in the habit of regarding as specially progressive.

If we add, that of the more than three and one-half millions of immigrants who came to our shores during the last decade the vast majority found homes north of Mason and Dixon's line, and accordingly contributed little or nothing to the growth of the Southern States, the tremendous recuperative energy of the latter section will be more fully appreciated.

The data with regard to the land area and density of population of the different divisions are interesting, but require no special comment.

The ratio of the sexes, displayed in the next section of the table, is entirely concordant with what an *a priori* judgment would lead us to anticipate. East of the Appalachian mountains, in the old settlements along the Atlantic coast, where social conditions have had time to crystallize into a permanent form, the sexes are practically numerically equal. West of these mountains, on the watershed of the Mississippi, where the settlement has been more recent, the males predominate, and this excess of males naturally increases the further west we proceed and the more recent the period of occupation. In the North Central division 32, in the South Central 20, and in the Western division no less than 124 males in each chiliad, or thousand, of the population are unprovided with female partners.

The proportion of the foreign-born population (supplied, of course, by immigration) presents in the next section some rather surprising figures. While in the North Atlantic, North Central and Western divisions the foreign-born constitute 16—23 per cent. of the total population, in the South Atlantic and South Central divisions the proportion of the foreign-born population is quite insignificant, never exceeding 2.5 per cent. The Southern States are occupied by a population almost purely native, and thus afford an ideal field for the study of the characteristics of native communities. At present the North Atlantic and New England States exhibit the largest proportion (22.6 per cent.) of foreign-born population, though the Western division follows closely with a proportion of 20.7 per cent. The general proportion of the foreign-born to the native population throughout the United States is 13.6 per cent., or about one to seven.

Table II., condensed from a similar table in Census Bulletin No. 103, affords some very interesting information relative to the amount and the character of immigration into the United States since 1820.

TABLE II.

| Countries. | Total | Number of Immigrants | | | | | |
|---|---|---|---|---|---|---|---|
| | | 1891—0[illegible] | 1881—90 | 1871—80 | 1861—70 | 1851—60 | 1821—50 |
| Aggregates in Chiliads | 19,115 | 3,688 | 5,247 | 2,812 | 2,315 | 2,598 | 2,456 |
| Canada and Newfoundland | 1,050 | 3 | 393 | 383 | 154 | 59 | 58 |
| Germany | 5,009 | 505 | 1,453 | 718 | 787 | 952 | 594 |
| Great Britain | 3,026 | 272 | 807 | 548 | 607 | 424 | 368 |
| Ireland | 3,869 | 388 | 655 | 437 | 436 | 914 | 1,039 |
| Norway and Sweden | 1,246 | 321 | 568 | 211 | 109 | 21 | 15 |
| Totals | 14,200 | 1,490 | 3,877 | 2,298 | 2,093 | 2,370 | 2,073 |
| Austria-Hungary | 1,027 | 593 | 354 | 73 | 8 | ........ | ........ |
| Italy | 1,040 | 652 | 307 | 56 | 12 | 9 | 5 |
| Russia and Poland | 929 | 602 | 265 | 52 | 5 | 2 | 1 |
| Totals | 2,995 | 1,847 | 926 | 181 | 24 | 11 | 6 |
| All other Countries | 1,920 | 351 | 444 | 334 | 197 | 217 | 376 |

It will be observed that the normal stream of immigrants for the preceding thirty years (1851–1881) was doubled in the decade 1881–90, and that a considerable part of this increase was also maintained in the last decade (1891–1900). Attention is specially directed to the sudden increase in the immigration from Austria-Hungary, Russia, Poland and Italy, begun during the decade 1871–80 and subsequently developed into such proportions as to rival in the last decade the immigration from all other quarters of the globe. The assimilative capacity of our people and our institutions is likely to be severely tested by this sudden irruption of alien races and alien civilization.

When, however, we come to study, in the next section of the table, the relations of color in the various divisions, we find the ratios just noticed practically reversed. In the northern and western States the negroes never quite attain 2 per cent. of the total population. In the southern States, on the other hand, the negroes constitute 30-36 per cent. of the population, and in Mississippi, *e. g.*, this proportion rises to 59 per cent., or considerably more than one half the total population. Such tremendous variations in the proportion of foreigners and of negroes in the different sections of our country necessarily imply differences of social condition, customs and habits, which demand extreme caution in criticism. Racial characteristics in miniature are of little importance. Aggregated in masses, they may not safely be judged on the grounds of pure theory.

An examination of the age-scale of the different grand divisions of the country reveals likewise differences of interest and importance. In the first place, we observe that the communal unit or chiliad of the northern divisions is perceptibly *older* than that of the southern. In the North Atlantic and North Central divisions, *e. g.*, we find respectively in each chiliad 592 and 544 adults. In the South Atlantic and South Central divisions, on the other hand, the adults in each chiliad number only 477 and 463. Conversely, the children and youths of the southern divisions number 523 and 537 to each chiliad, while in the northern divisions 408 and 456 are the corresponding figures. Adults form, therefore, the majority of northern communities; children of southern.

Again, each chiliad of the North Atlantic division exhibits 108 children under the age of five years. The same unit in the North Central States displays 116 children of the same age. But when we compare with these the figures of the southern States, we are surprised to find the latter largely increased, the South Atlantic division exhibiting 140 children under five years of age to each chiliad, and the South Central no less than 144 children of like age to the same unit. In other words, the southern communities contain about one-third more young children than their northern associates, and about one-fifth more youths between the ages of 5 and 20 years. These are startling differences, and their cause is worthy of careful examination.

Three hypotheses present themselves for investigation in this connection: 1. The heavy influx of immigrants into the northern divisions, and the fact that the majority of these immigrants are young unmarried adults, may produce a fictitious appearance of unproductiveness in the adult population of the States chiefly affected by immigation.

If this is the true explanation of the disparity in the numbers of children of the contrasted sections, we may naturally expect to find a similar disparity in the number of marriages of the same sections. Unfortunately for our purpose, the tables recording the conjugal condition of our population in 1900 are not yet published. It is, however, reasonable to expect that they will not differ very materially from those of the census of 1890. According to the latter, the number of females in each chiliad of our population who in 1890 were or had been married (married, widowed or divorced) was, in each of the grand divisions of our country, as follows:

| | |
|---|---|
| North Atlantic division | 232 |
| North Central division | 213 |
| South Atlantic division | 301 |
| South Central division | 201 |
| United States in general | 210 |

We are somewhat surprised to find the relative frequency of marriage the greatest, precisely where the proportion of children to adult population is least.

II. It may be argued again that the predominance of children in our Southern population is due to the large percentage of negroes in these communities, and to the general licentiousness of a race comparatively indifferent to the obligations of the marriage vow. Table III. furnishes us a comparative age-scale of the white and negro communities in the South Atlantic and South Central divisions of the United States.

TABLE III.

| Age. | South Atlantic. | | South Central. | |
|---|---|---|---|---|
| | Whites. | Negroes. | Whites. | Negroes. |
| 0—4 | 134 | 148 | 140 | 155 |
| 5—20 | 369 | 409 | 385 | 406 |
| 21 plus | 497 | 443 | 475 | 439 |
| Total | 1,000 | 1,000 | 1,000 | 1,000 |

It is evident from this table that the proportion of children to adults is, indeed, consideraly larger among the negro population of the South than among the whites of the same section. But a comparison of this table with the age-scale table I. will show likewise that the proportion of children to adults among the *white* population of the South is considerably larger than among the same class of the Northern States. Each chiliad of the North Atlantic division of States, *e. g.*, is represented by 108 children under the age of five years. But a chiliad of the white population alone in the South Atlantic States exhibits 134 children under the same age, an increase of almost 25 per cent. Nearly the same difference is noticed in the child population of the North and South Central divisions of the country. We are accordingly driven to the acceptance of a third hypothesis, to wit: that the fecundity of the Southern white population (and *a fortiori* of the mixed population of the South) exceeds very considerably that of the general population of the Northern States. This conclusion is also supported by the figures of the census of 1890, showing the number of persons to a family in each division of the United States for the last three decades.

TABLE IV.

| | Individuals to a Family. | | | |
|---|---|---|---|---|
| | 1890 | 1880 | 1870 | Mean. |
| North Atlantic | 4.69 | 4.80 | 4.92 | 4.80 |
| South Atlantic | 5.25 | 5.19 | 5.17 | 5.20 |
| North Central | 4.86 | 5.12 | 5.23 | 5.07 |
| South Central | 5.30 | 5.25 | 5.18 | 5.24 |
| Western | 4.88 | 4.75 | 4.37 | 4.67 |

It will be observed that the average family of the Southern divisions exceeds considerably that of the Northern, and further that, while in the Northern divisions the size of families has been gradually decreasing for the last thirty years, in the Southern divisions there has been an equally persistent increase for the same period.

If we desire to convert the data of these age-scales into approximate birth-rates, the latter may be arranged as follows:

TABLE V.

| Division. | Birth Rate. |
|---|---|
| North Atlantic.................................... | 28.5 per 1,000. |
| South Atlantic.................................... | 37 " " |
| North Central.................................... | 30.5 " " |
| South Central.................................... | 38 " " |
| Western.......................................... | 27.7 " " |
| United States .................................... | 32 " " |

An examination of the figures of this table explains the rather surprising rate of increase of the population of the Southern States during the last decennium. It is to be remarked also that this growth was rather intrinsic than extrinsic, the result of the vital energies of the people themselves, and in no considerable degree dependent upon foreign immigration.

The increase of the population of the Southern States would have been even more marked, were it not that the increased birth-rate of this section was largely counterbalanced by an increased mortality in all classes, though of course largest among the negroes. Table VI. contrasts the mortality of six Northern and six Southern cities, selected merely for their close correspondence in the number of their population.

TABLE VI.

URBAN MORTALITY.

| Northern Cities | Population | | | Mortality. | | | Rate per 1,000 | | |
|---|---|---|---|---|---|---|---|---|---|
| | White | Colored | Total | White | Colored | Total | White | Colored | Both |
| Detroit, Mich..... | 281,575 | 4,129 | 285,704 | 4,790 | 103 | 4,893 | 17.0 | 24.9 | 17.1 |
| Jersey City, N. J.. | 202,510 | 3,923 | 206'433 | 4,177 | 100 | 4,277 | 20.6 | 25.5 | 21.1 |
| Fall River, Mass.. | 104,458 | 405 | 104,863 | 2.341 | 4 | 2,345 | 22.4 | 9.9 | 22.4 |
| Cambridge, Mass.. | 87,875 | 4,011 | 91,886' | 1,598 | 101 | 1,699 | 18.2 | 25.2 | 19.3 |
| Dayton, Ohio..... | 81,923 | 3,410 | 85,333 | 1,333 | 72 | 1,405 | 16.3 | 21.1 | 16.5 |
| Camden, N. J..... | 70,288 | 5,647 | 75,935 | 1,069 | 166 | 1,235 | 15.2 | 29.4 | 17.6 |
| Total........... | 828,629 | 21,525 | 850,154 | 15,308 | 546 | 15,854 | 18.5 | 25.4 | 18.6 |
| Southern Cities | | | | | | | | | |
| New Orleans, La.. | 209,390 | 77,714 | 287,104 | 4,977 | 3,310 | 8,287 | 23.8 | 42.6 | 28.9 |
| Louisville, Ky.... | 165,592 | 39,139 | 204,731 | 2,970 | 1,122 | 4,092 | 18.0 | 28.7 | 20.0 |
| Memphis, Tenn... | 52,410 | 49 910 | 102,320 | 1,145 | 1,427 | 2.572 | 21.8 | 28.6 | 25.1 |
| Atlanta, Ga....... | 53,960 | 35,912 | 89,872 | 1,250 | 1,137 | 2,387 | 23.1 | 31.7 | 26.5 |
| Richmond, Va.... | 52,820 | 32.230 | 85,050 | 1,294 | 1,229 | 2,523 | 24.5 | 38.1 | 29.7 |
| Nashville, Tenn.. | 50,821 | 30,044 | 80,865 | 1,055 | 987 | 2,012 | 20.8 | 32.9 | 25.2 |
| Total.......... | 584,993 | 264,949 | 849,942 | 12,691 | 9,212 | 21,903 | 21.7 | 34.7 | 25.8 |

It will be seen that while the *general* mortality of the Southern cities is nearly 40 per cent. larger than that of their competitors in the Northern States, the mortality of the *white* population of the Southern cities is much less excessive, averaging only about 17 per cent. above that of the Northern cities compared. The formidable death-rate of our Southern cities is due chiefly to the negro population, whose mortality, it will be observed, is high, even in the cities of the North. With our present increased knowledge of the causes of epidemic disease and of the sanitary measures required for its prevention, it may be hoped that much of the avoidable mortality of the Southern cities may be eliminated, and that they may assume in a few years the sanitary position to which their delightful climate and often pleasant location may fairly entitle them. What may be accomplished in this direction can be judged from the statistics of Louisville, Ky., whose death-rate in 1900 exceeded but little that of the average Northern city.

The net results of the factors thus studied upon the growth of the various sections of the country may be roughly stated as follows. The North Atlantic, North Central and Western divisions, with a population in 1890 of 42,919 chiliads, increased in the decennium 1890–1900 by 4,864 chiliads (excluding immigration), or 11.3 per cent. The South Atlantic and South Central divisions, with an initial population of only 20,027 chiliads, gained in the same decennium a population of 4,496 chiliads, or 22.4 per cent., almost exactly twice the rate of increase of their more populous Northern rivals. That a not insignificant proportion of the gain of the Southern divisions was derived from the North is undoubted, but we have at present no data upon which to base an estimate of its magnitude.—*Cleveland Medical Journal.*

---

### HOW TO CONDUCT A NORMAL LABOR.*

We, as physicians, ought to give more care to the pregnant and puerperal woman than we usually do.

As soon as I am engaged to attend a woman and have made a

* Read at the meeting of the New York Celtic Medical Society at the Academy of Medicine, in February, 1902, by James Moran, M.D., New York, Instructor in the Department of Gynecology in the New York School of Clinical Medicine; Member of the Medical Society of the State of New York, the New York State Medical Association, the New York Celtic Medical Society, and the American Medical Association.

diagnosis of pregnancy, I examine the urine, and have her bring once a month a sample of the night's and morning's water. After the eighth month I examine it every week, particularly if it contains albumin, sugar or tube-casts. I examine the heart and lungs also, and give my patient instructions in regard to diet, exercise and clothing.

The diet should be of a nature easily digested and nourishing in character. A moderate share of exercise daily in the open air is very necessary to the health of the pregnant woman. Violent exercise and long journeys over bad roads should be avoided if possible. Frequent or daily baths of moderately warm water help to keep the skin active and the patient in good condition. The bowels should be kept regular, if necessary, by a mild cathartic.

The clothing should be worn loosely and hung from the shoulders, so that there may be no constriction around the waist. The circular garters should be replaced by the long side supporters. Corsets, if worn at all, should be worn loosely, and after the fifth or sixth month discarded and a corset-waist used instead. Wearing of tight corsets predisposes to mal-development and mal-position of the fetus, and also impedes the functions of the kidneys. The breasts if pendulous should be supported by a suitable bandage or underwaist. The nipples should be kept free from pressure. Any mild ointment may be used to keep them soft and in a healthy condition. If depressed they should be massaged and manipulated to bring them into proper shape.

On the first or second visit of the patient to my office I get her history as far as possible. If she has had children, and difficulty in previous labors, I try to ascertain the cause and nature of the difficulty, whether it was due to kidney disease, tumors, deformed pelvis, or very large children. With a primipara I examine to ascertain if there is any deformity or mal-formation or any diseased condition. If the trouble in the multipara is due to a small pelvis or a very large child, I can do much to lessen the difficulty in the next confinement by limiting the amount of food and drink of the mother during the last two months.

I allow her very little liquid during the last month, provided there is no trouble with the kidneys. By this method the child will not accumulate a lot of unnecessary fat.

*The Room and Bed.*—I give each patient, when she comes to engage me, a printed card with instructions as to the proper kind of room and how to prepare the bed, and a list of the things that are necessary for herself and the child.

The patient should be confined in a large and well ventilated room, and a sunny one if possible. If the weather is cold the room should be kept warm by steam, gas or a stove. The room should be well aired and cleaned before admitting the patient, and if there has been any infectious disease in the room a thorough fumigation is necessary.

The bed should have a firm mattress, covered with rubber or oilcloth, a clean sheet or blanket should next be put on over the rubber, and both fastened to each side of the mattress and at the corners with safety-pins. Then on top of these where the patient lies should be placed a second piece of oilcloth, four feet square, and over this a pad or clean sheet, folded three or four times. The bed is now ready for the patient. A rug or oilcloth is placed at the bedside to protect the carpet.

*Preparation of the Patient for Labor.*—She should have much the same care and treatment as if she were undergoing an operation. When the pains of labor are felt some medicine should be taken to move the bowels. Either of the following will suit: (1) Epsom salts, one tablespoonful in a cup of hot water; or, (2) a seidlitz powder in a cup of water; or a bottle of magnesia.

Then, two or three hours later, an enema should be administered of a quart of hot water and soapsuds, with one drachm of spirit of turpentine; the injection should be repeated every half-hour until the bowels move freely. The patient should then take a warm bath, using plenty of soap and washing the genitals with great care, after wiping dry. She then puts on a clean chemise or night-dress, and a clean sheet is now folded once across and put around her waist and fastened in front with a large safety-pin. The night-dress can then be folded up above the sheet and fastened so as to remain there until after delivery. The patient should not use the water-closet after this, but the chamber or bed-pan.

Vaginal douches I do not give unless there is some infection of the genital tract. I make sure that the bladder is emptied during the first and the early part of the second stage of labor.

Little or no solid food should be given after labor begins. Solid food in the stomach might be very injurious should surgical anesthesia be required. Chicken broth, mutton broth or other nourishing soups and plenty of water may be given during the first stage of labor.

*Outfit for Patient and Child.*—One rubber sheet or oilcloth large enough to cover the entire width of the bed and the greater part of its length. A second piece of oilcloth about four feet square. Six abdominal binders one yard and a quarter long and half a yard wide, made of the cheapest grade of unbleached muslin. Four bed pads, each four feet square and two or three inches thick, made of cheese-cloth and stuffed with non-absorbent cotton. They should be stitched and tufted enough to prevent the cotton slipping. One pound of absorbent cotton. Twenty-four vulvar pads or napkins, sterilized. Twenty-four baby napkins. One small blanket to wrap the baby in. Three-quart or four-quart fountain syringe and a douche-pan. Two or three wash-basins and from half a dozen to a dozen clean towels. From six to twelve clean sheets. Rug or oilcloth to protect the carpet at the bedside. Eight ounces of olive oil and a cake of ivory or castile soap. One new nail-brush. Two dozen large and two dozen small safety-pins. Three clean aprons. One pot or kettle of boiling water and a pot of cold water, boiled and covered with a towel.

*Examination of the Patient.*—Before making the examination I scrub my hands and arms for several minutes with soap and water, changing the water two or three times, then soak them for a few minutes in a solution of bichloride of mercury, then rinse them off in a two per cent. solution of lysol. A similar solution of lysol is used to wash off the patient's genitals just before the examination is made.

I never make an examination under cover of the bed-clothes or the patient's clothing, as contact with any of these may infect the examining fingers. I use sterilized rubber gloves if I have attended any infectious cases a short time previously.

In my first examination of the patient I try to ascertain the condition of the vagina and cervix, the position and presentation and the probable duration of labor, and whether or not it will be necessary for me to remain. I do not make frequent examinations.

All this care and preparation of the patient and washing and scrubbing with the use of antiseptics may seem unnecessary and impossible to carry out, particularly with a poor patient in a tenement house. But I find that almost all of it can be done with very little difficulty if the physician takes the trouble to impress on his patient the necessity of thorough cleanliness and the great danger of sepsis or childbed fever, and most women feel their importance more when there is some fuss made over them, and will take extra efforts to have something that their neighbor never had for a confinement.

I must confess that I did not take these precautions until the last few years. During my first years of practice I had several cases of puerperal fever—some running a mild course, others with a high fever for two or three weeks, and three patients died of sepsis, contracted no doubt in labor. My experience in this misfortune is no exception to the general rule, as I have seen many such cases in consultation in the practice of other physicians.

When I take all these precautions I find I have no trouble with my confinements. They run through a normal temperature, and I am saved a lot of worry and all the trouble I formerly had in washing and curetting the uterus. Now, I have no trouble with the so-called milk-fever and a "touch of malaria," that formerly served as a cloak to cover my mistakes.

In addition to the usual things in my obstetrical bag, I also have a double tenaculum, a uterine dressing forceps, and a square yard of iodoform gauze in a sealed glass battle, so that in case of post-partum hemorrhage, I can pull down the cervix and pack the uterus. I also carry a needle-holder and perineum and cervix needles with sterilized sutures, so that I can repair all lacerations at once.

When the pains are severe and the os is rigid, I usually give twenty grains of chloral every half-hour, till three or four doses are taken. This may be given by either the mouth or rectum.

Quinine and sulphate of strychnin I find act better than ergot in producing uterine contractions and keeping up the tone of that organ, and there is not the danger in using these that there is in the use of ergot. Morphine, in one-eighth grain doses, I use now in almost all my cases; where the woman is suffering severely and labor is progressing slowly, I give it every hour and sometimes every half-hour. It does not prolong labor, but allows the woman to bear down with more force and very much less suffering, and it calms her fears. It gives me often a chance to go and make two or three calls before she requires my assistance, and it acts much as does chloral in relaxing the cervix and perineal muscles.

I find most patients do better by being on their feet until labor is well advanced. When the patient goes to bed, I place under her hips the Kelly pad, covered with a clean napkin. This saves the bed from the discharges.

When the child's head is well down and pressing on the perineum I give a little chloroform during the pain. I find that this enables her to bear down with more force and less suffering. It also causes relaxation of the cervix and perineum. When the occiput is well down under the pubic arch and the vulva wide open, the patient's limbs should then be kept extended, in order to relax the perineum as much as possible. When the knees are flexed upon the abdomen, the skin of the perineum is put on the stretch, and there is more danger of it tearing than when the legs are extended. Sufficient chloroform may now be given to stop pain and muscular action. I can now usually, by upward pressure on the child's forehead, deliver it in from fifteen to twenty minutes. This method will save the perineum from tearing, in the great majority of cases, and spare the mother much keen suffering. As soon as the head is born I stop the chloroform and wipe off the child's eyes with a saturated solution of boric acid.

In cases where labor is progressing very slowly and the pains are nagging in character and of very little force, if the cervix is well dilated, I find by experience that it is better to use the forceps, and, by slow and easy traction, deliver the child. In such cases waiting too long wears out the strength of the mother; the uterus loses its tone and power of contraction, and there is great danger of post-partum hemorrhage.

Following the delivery of the child I make firm pressure on the fundus, and this pressure is kept up by myself or the nurse for at least half an hour after expulsion of the placenta, or until all danger of hemorrhage is passed. After the placenta is delivered it should be examined carefully to see that no part of it remains in the uterus.

All blood-clots should be removed, and a careful examination of the vagina and cervix made, and any lacerations found should be repaired at once if the patient's condition will permit it. A sterilized gauze pad is then placed over the vulva and the abdominal binder is put on as soon as the uterus is firmly contracted. The binder should be fastened from above downward and the uterus should be pressed firmly against the pelvic brim. Putting on the binder from below upward may push the uterus into the abdominal cavity, causing it to relax and produce hemorrhage. The patient should now be put in bed and have absolute rest, and friends and relatives be kept out of the room.

After an hour or two, if the mother is in good condition, the child can be put to nurse; this is done to stimulate uterine contraction and to increase or hasten the flow of milk. Before the child is put to nurse, its mouth should be washed with a solution of boric acid; the mother's nipples should be washed at the same time with a similar solution, and this should be repeated after each nursing. The patient should remain quietly on her back until all danger of hemorrhage has passed. After the second day I advise my patient to lie on the abdomen for a short period of from five to twenty minutes, several times each day and night while she remains in bed. This is done for two reasons: First, to prevent or correct a retroversion or retroflexion, which may have previously existed; secondly, to afford better drainage from the vagina.

The diet of the mother, after labor, should be liquid: milk, cocoa, soups, and plenty of water, may be given to stimulate the kidneys and bowels. After the second or third day a little solid food may be given. So long as there is a free reddish discharge the patient should be kept in bed, except when she has to get up to attend to the calls of nature.

When I make my last call, which is usually about the tenth or twelfth day, I make a careful examination of the vagina and uterus, to ascertain the condition, and I give instructions as to douches or any other treatment that I may see indicated.

Before leaving my patient I impress on her the necessity of nursing the child, no matter how difficult it may be, and warn her of the great mortality of infants artificially fed, and I instruct the mother as to the proper kind of diet to use, in order that she may have sufficient good milk to nourish her child. Water should be drunk freely between the meals, and, by so doing, constipation of both mother and child is prevented.—*Dominion Med. Monthly.*

---

### EXAMINATION OF BODIES FOUND DEAD IN WATER.

The problem, which in some instances is a very intricate one, of the cause of death of a person whose body has been found in the water has come up for solution in the case of Miss Nell Cropsey, a young lady of Elizabeth City, N. C., who very mysteriously disappeared from her home on the night of November 20th last, and who was seen no more till thirty-seven days afterwards, when her dead body appeared floating in the river not far from her residence. The last known of her alive was about 11 o'clock that night, when she was on the piazza of her house, whither she had gone from the parlor with her lover, James Wilcox. They were at variance at the time, but their disagreement was regarded as a mere lovers' quarrel of no moment. About 12:30 o'clock she was missed, and search for her was made. Wilcox was found at his home in bed, and when told of the disappearance of Miss Cropsey he rose and went to her house. Here he stated that he had left her on the piazza crying, and solemnly averred that he did not know where she was. Nevertheless, it was suspected that he had murdered her, and his subsequent demeanor, which was very sullen and unfeeling, contributed much to strengthen the suspicion. Accordingly, he was arrested, and has recently been tried and convicted of the crime.

In a case of this kind, where the available facts are few and not very cogent, the examination of the dead body is manifestly a resource of extreme importance. We regret that we have been compelled to obtain our knowledge of the procedures in the examination of Miss Cropsey's body entirely from newspaper accounts, having the inevitable imperfections incident to reports of technical matters by inexpert reporters. These accounts are not only very inaccurate and incomplete, but much of them is presented in such a confused manner that it is scarcely intelligible to us. Moreover, the contending lawyers, religiously obedient to the principles and practice of their art, which require them to assiduously quench any glimmer of adverse truth that may chance to be straggling through the encircling gloom, have done their best, and have measurably succeeded in it, to befog everything by muddling up the facts and harrying the witnesses into a state of obfuscation. All of this is, of course, common enough, being exceedingly likely concomitants of any case that is reduced to so forlorn a condition as to be dependent on our so-called courts of justice for unraveling it and eliciting its facts as they actually and truly are, and we mention it only to secure ourselves should we misstate anything or do wrong to any one.

Miss Cropsey's body was found floating about seventy-five feet from the bank of the river and a few hundred feet from her house. The examination was commenced about an hour after the body had been taken out of the water. The jury was permitted to be present during the examination, a procedure which, in our opinion, is not an advisable one, but which in this instance seems to have been advantageous, as will appear further on. Her clothing had not been injured or disarranged, and there were no marks of violence that were immediately obvious. Externally there were no decided indications of decomposition except that the hair and scalp were loose and peeling, and internally the brain, whose condition is described as "defluent," was the only organ decomposed. The genitalia were those of a virgin. The stomach and intestines were normal. No water was found in the stomach; in fact, the organ was practically empty. There was no blood in any of the heart cavities. The examination of the lungs appears to have been restricted to cutting off a piece of the right lung, squeezing it, and noting the extruded matter, which was in small amount and in the form of small bubbles of black, bloody froth. The air passages were not examined. No water was discovered in the lungs or pleural cavities.

At this stage the physicians decided to end the autopsy, but on reporting their results to the law officer he directed them to make a further examination. Accordingly, they proceeded to examine the head. There was no external indication of injury except a small swollen place on the left temple. This could not have been very obvious, for it had escaped the notice of the medical men, and was first perceived by one of the jurymen; whose observational faculties had perhaps been sharpened by the exercise of his profession, which was that of a barber. Its size is stated as about two inches by two inches. On cutting into it there was found a collection of dark fluid blood, of circular form, and of about a tablespoonful in quantity. The pericranium beneath exhibited a slightly blue discoloration. No effusion of any kind was seen on or in the brain, this organ, it seems, being considerably decomposed.

At the trial the medical experts testified that, in their opinion, Miss Cropsey did not die from drowning, but that her death was due to a blow on the head, inflicted with a heavy round covered or padded instrument. That she did not drown they thought was shown by the absence of water from the lungs, pleural cavities, and stomach, admitting, however, that it was not conclusively shown, because of the fact that, in a large percentage of cases of drowning, water is not found in these parts. The lawyers for the defence claimed that the absence of water from the stomach and lungs could be accounted for by osmotic action, and much erudite talk was bestowed on this interesting process, with the result that one of the medical witnesses was led to admit that this might be so, though what had become of the transuded water was not made manifest.

As well as we can ascertain from the reports we have of the trial, the appearances on the head were, without question, conceded to be marks of violence. Yet that they might be owing to putrefactive changes seems to us to be at least a plausible supposition, and it is surprising that the lawyers for the defense did not seize on this notion and maintain it to the death. The defense appears to have been conducted on the theory of suicide, and color was given this view by a conversation on suicide held in the parlor on the night of Miss Cropsey's disappearance, in which she took part; but this was apparently a mere off-hand chat, and, indeed, it happened that during the conversation Miss Cropsey expressed herself as opposed to drowning because of its disfiguring effect upon the hair. Besides, she knew how to swim—a circumstance which, we think, makes it unlikely that she would choose drowning as the means for ending her life.

As is usual in this class of cases, medical writings were made to cut a distinguished figure, and the medical witnesses were held to owe unquestioning allegiance and to be under absolute subjection to the consecrated scribblers. One of the witnesses, in the course of his rummaging by the hostile lawyers, was bamboozled into sanctioning their pretended belief in the infallibility of certain so-called authorities, and naturally got into trouble thereby. To give this sanction is not only often very unsafe for a witness, but it is, besides, not justifiable. Those who are obliged to have much to do with "authorities" know very well that some of their statements are altogether wrong, and that many of them must be materially modified in order to express the facts correctly. Undoubtedly the authorities should be treated with respect and reasonable deference, but assuredly it is not incumbent on us to give up the established results of our own observation and experience to their dicta, nor to unreservedly adopt the estimate of them which a lawyer, for the time and for a purpose, strives to force upon us.

The case of Miss Cropsey presents numerous features of great medico-legal interest and importance, but as it is still in the courts awaiting a final decision we deem it inexpedient to discuss them at this time.—*Old Dominion Journal of Medicine and Surgery.*

---

### CHILDREN'S WEIGHT AND HEALTH.

Of late years there has come the realization that the body weight represents one of the most significant signs of the general health of the individual. Most of the large life insurance companies prefer a risk on a life handicapped by serious family heredity rather than one on an individual who is twenty pounds under the average weight for his size and age. Of course, the medical significance of the body weight does not necessarily imply that in order to be healthy an individual must come up to any arbitrary standard, but that, with due allowance for family antecedents in this matter, he must not fall far below the known average weight for his height. If this is true of adults, it is, if possible, even more significant in children. The weight of the child, and its increase, constitutes the best index of health and healthy development not only during infancy but also during later childhood.

Little account is usually taken of the child's weight from month to month. Very seldom is the family physician able to obtain anything like a continuous record at regular intervals. Such a record would surely reflect the child's health during its developmental stage better than anything else. Plenty of esoteric information is usually injected into the mother's account of the child's health. Subjective symptoms of the ordinary variations of feeling, due to the weather, to passing constipation, or to deserved indigestion, are retailed to the physician, when the child finally comes down with some illness. The one record of objective conditions that is easiest to keep and that is most significant—the body weight at regular intervals—is, as a rule, utterly deficient. It must be conceded that one reason for this is that physicians generally have not appreciated the help such a record might be in furnishing data for the study of the course and progress of the child's development, its interruptions, its phases of more or less stagnancy and its times of acme.

The careful notation of the child's weight is especially valuable during the years that immediately precede and follow puberty. It is at this time that the strains and stresses of life are most serious. Not infrequently, when the facial appearance would seem to proclaim a condition of excellent health, the body weight will be found to negative the conclusion that might be drawn from the child's appearance. This loss of weight will often serve to account for the lassitude, disinclination to study and capricious appetite that are sometimes set down to laziness.

The unfavorable attitude of many children towards fats is very well known. Often this distaste is found to exist in just those who need most to have fats liberally supplied in their dietary. Sometimes the fact that one or both parents have had similar feelings at a corresponding age is taken to mean that the distaste is hereditary, and only too often induces the unwarranted assumption that nothing can be done for the condition except to make up for fats by starchy substances. As a matter of fact, what is mainly needed is judicious selection and careful preparation of the fats to be taken. Cream, and the vegetable oils particularly, may, with a little tact, be made to enter into a child's dietary to its decided advantage and without exciting disgust. Even crisp bacon and good butter may also be made to supply the fat needed in these cases if tastiness is properly consulted.

Where, notwithstanding judicious selection of materials, not as an incidental experiment, but as a continuous direction for some time, the child fails to come up to normal weight, or where, because of the stress of intellectual work, it drops below the standard at times of special strain, this symptom must be the indication for the exercise of special watchfulness and the positive cessation of extra work. How often, about examination time, do children blanch, grow pinched and thinner than before. Nothing much more serious than this could happen during the precious development period. The young plant would never stand the strain of wind and weather changes if it had to stand up before them. To make children precocious is not to foster greatness later in life, but always the reverse.

Physicians should impress upon parents the necessity for regarding failure to reach the average weight as a sign that children should not be pushed or even encouraged to do serious work in early life, and to consider loss of weight during the years before puberty as a positive danger signal. The standard given by Hope and Browne in their recently issued "School Hygiene," forms a ready guide. At five years of age a child should weigh about as many pounds as it is inches high. This is about forty. If sturdy it should weigh a pound or two more. The rate of increase should be about two pounds per inch of growth, with a tendency for the proportion of weight rather to increase. Excess of weight in proportion to height is a favorable sign, while a deficiency in weight is unfavorable. For the child to be much below this standard is to subject it to the risk of every infection that may happen its way, for its resistive vitality is below par. For the child to drop from its normal weight because of over-pressure at school should be the signal for the physician to insist on the active intellectual work being given up. When children are growing rapidly, and their height-weight ratio is disturbed, they should have long hours of rest, at least nine hours at night and an extra hour in the daytime, and all fatiguing occupations, even games, should be interdicted for a time. The developmental period is too precious for the after-life, and any possible intellectual benefit of too little significance to allow trifling in this matter. Mental training may be compensated for later, but physical development never; and the weight constitutes a ready and easily recognized index of how much may be allowed so that one shall not interfere with the other.—*Archives of Pediatrics.*

---

### THE SARATOGA MEETING OF THE AMERICAN MEDICAL ASSOCIATION.

The fifty-third annual meeting of the American Medical Association was a disappointment in only one respect, the fact that the number of members registered, 1,410, is less than the attendance at any other recent meeting. There were, however, a much larger number of unregistered visiting physicians in attendance at the meeting than ever before.

The general meetings, the sessions of the House of Delegates and the short business meetings of the various Sections were all characterized by unusual harmony. The sessions of the Sections began promptly on schedule time, but with the exception of the final meeting not one of the general meetings began on program time. The time for the general opening of the meeting was changed from ten to eleven, and many did not know of the change, nor did it open until some time after eleven. The evening sessions opened more than half an hour after published time, and not a few members and ladies left the convention hall each night before the exercises opened, going to some of the many other attractions.

The social features, planned to interfere as little as possible with the scientific work, were varied, and the members and ladies found their stay in Saratoga most delightful. The receptions, charming drives and excursions were greatly enjoyed. The wearers of badges, furnished the members and their ladies, received many favors and courtesies. Mention should be made of the reception given to members and ladies Wednesday afternoon by Mr. and Mrs. Spencer Trask, at their beautiful villa, "Yaddo," and the all-day excursion on Thursday to and on Lake George, including dinner at Silver Bay House.

The officers of the Sections ought to be congratulated upon the variety and value of the practical scientific papers read. There could not have been a member who would not have gained satisfaction and profit by attending during the whole week Sections other than the one in which they had registered.

The new constitution, adopted last year, provides for the transaction of all business in the House of Delegates. Pennsylvania was entitled to the largest delegation, and all of her eight delegates were present. The House was composed of decidedly representative members of the profession, and only one State was not represented. The body is still large for the ready dispatch of business, and it may become necessary to change the basis of representation. It is evident, however, that the House of Delegates is a decided improvement upon the previous method of transacting the business of the Association. At least one-half the members of the House this year will be members of next year's House, and the experience and organization gained this year will still further facilitate the satisfactory transaction of business next year. The entire country was divided into nine districts, with a representative from each district, selected by the members from the district on the nominating committee. The ninth district, consisting of New York, Pennsylvania, New Jersey, Maryland and Delaware, was represented by Dr. W. S. Foster, of Pittsburg. Dr. Foster was also appointed a member of the Business Committee, which holds over for next year. Dr. W. T. Bishop, of Harrisburg, is chairman of a committee to revise and correct the list of members of the Association. Dr. J. B. Roberts, of Philadelphia, was appointed a member of the Finance Committee.

Dr. E. E. Montgomery, of Philadelphia, was reelected a member of the Board of Trustees, and Dr. J. M. Anders, of Philadelphia, was elected to give the Oration in Medicine next year.

Dr. Frank Billings, of Chicago, was elected President, and St. Louis was selected as the meeting place for 1903. A committee was appointed to consider the matter of the Revision of the Code of Ethics.

A disastrous fire broke out early Monday morning, causing the death of five persons and the loss of $300,000 worth of property. The United States post-office, the telephone office, the plant of the principal daily paper and several rooms intended for use of various Sections were burned. The mails were necessarily destroyed, and all that was in the office at the time was delayed. The plans that had been perfected for reporting the Association news and work were rendered inoperative. The Saratoga meeting will long be remembered as one of the best in the history of the Association, and the thanks of the membership are due to the New York State Association, the Saratoga physicians and the officers of the Association.—*The Pennsylvania Medical Journal.*

---

### THE PREVENTIVE AND CURATIVE TREATMENT OF HAY FEVER.

It is difficult to conceive of a more miserable creature in all the world than the hay fever sufferer. The attack not only makes him exceedingly uncomfortable, but renders him unfit for business or the pleasures of society. Aside from the annoying and continual discharge from the nostrils, the eyes are suffused, the secretion of tears is increased, the nasal passages are obstructed, and an intense burning sensation is experienced; the latter is not entirely limited to the mucous membranes, but not infrequently involves the cutaneous surfaces of the forehead, cheeks and nose. Violent attacks of sneezing occur which are so prolonged, at times, as to completely exhaust the sufferer and bring on severe headache. The condition is one of utter wretchedness, and there is extreme malaise, amounting occasionally to complete prostration. The lightest duties become irksome tasks, and many an active, industrious, and useful member of society is completely incapacitated while "the season" lasts.

For years some convenient means of relief has been sought. Change of scene does very well for those, unfettered by business, who can afford to travel. But to many very worthy people a change of scene is out of the question. Naturally the greater number of the afflicted are accustomed to look to the medical profession for the help they need. But what has the medical profession actually accomplished for the permanent relief of the sufferer or the cure of his ailment? There is scarcely a sedative, astringent, tonic, nervine, or alterative drug in the materia medica that has not enjoyed an evanescent reputation as a useful remedy in the treatment of hay fever. Until the discovery of Adrenalin, each had been as much of a disappointment as its predecessor and none had afforded more than the merest temporary relief.

There is increasing evidence that Adrenalin fully meets the indications as a remedial agent in hay fever. It controls the nasal discharge, allays congestion of the mucous membranes, and in that manner reduces the swelling of the turbinal tissues. As the nasal obstruction disappears, natural breathing is materially aided and the ungovernable desire to sneeze is mitigated. In short, a season of comparative comfort takes the place of the former condition of distress and unrest. Adrenalin blanches the mucous membrane by vigorously contracting the capillaries, and thus reduces local turgescence. It strengthens the heart and overcomes the sense of malaise so frequently a prominent feature in cases of long standing.

In the treatment of hay fever the Solution of Adrenalin Chloride should be used. This preparation is supplied in the strength of one part Adrenalin Chloride to one-thousand parts Normal Saline Solution, and is preserved by the addition of

0.5 per cent. Chloretone. The 1-1000 solution should be diluted by the addition of four parts Normal Salt Solution, and sprayed into the nares with a "Cocaine" atomizer. In the office, the 1-1000 solution may be applied in full strength. A small pledget of cotton is wrapped about the end of an applicator and moistened with a few drops of the solution (1-1000). The speculum is then introduced, the patient's head is tilted backward in a position most favorable for thorough illumination by the head-mirror, and the visible portions of the lower and middle turbinate bodies, and the septum, are carefully and thoroughly brushed. The same application is made to the other nostril, when usually relief follows, in a few moments. Should the benefit prove only partial, the 1-5000 solution may now be sprayed into both nares, and a few drops instilled into both eyes. The effect of this treatment may be expected to last for several hours. Indeed some physicians report that it is necessary to make but one thorough application daily to afford complete relief.

It is also recommended that Solution Adrenalin Chloride be administered internally in five- to ten-drop doses, beginning ten days to two weeks prior to the expected attack. In explanation of the beneficial effect of the drug when used in this manner, the suggestion has been made that hay fever is essentially a neurosis, characterized by a local vaso-motor paralaysis, affecting the blood supply of the eyes, nose, face and pharynx, and occasionally of the laryngeal and bronchial mucous membranes. Adrenalin overcomes this condition, restores the normal balance in the local blood pressure, and thus aids in bringing about a cure. The profession is to be congratulated that it has at last an agent that, if not a specific, fulfills the therapeutic indications more completely and with greater satisfaction than any other remedial measure recorded in the history of medicine.

---

### SURGERY OF THYROID GLAND.*

After reviewing the human and comparative anatomy (microscopical and topographical) many assigned causes are given, the

* Abstract of a paper read before Ohio State Medical Society, Toledo, O., May 27, 28 and 29. Illustrated by fifty lantern slides, by B. Merrill Ricketts, M.D., Cincinnati.

most common being heredity, acute infectious diseases and malignant neoplasms. All vertebrates are subject to the same laws concerning disease and abnormalities of the thyroid gland. Nephritis from any cause is a common cause, and when it is a cause the growth is of rapid development. Thyroiditis is rare and when present follows an operation or injury. Of the parasites, echinococcus and cysterous are rare causes, while the bacilli of pneumonia, typhoid and tuberculosis and micro-organisms of a selective type are more frequent. The results of disease of the thyroid gland are insanity, infection, hemorrhage, dyspnea and rupture. Death may be due to any one or all of these causes. The thyroid gland is subject to nearly all forms of benign and malignant neoplasms. The treatment is classified as: 1. Medicative. 2. Operative. Medicative is of but little avail except to palliate. Extracts will benefit but not cure. They will lessen the size of the neoplasm. Only commendable in a certain class of cases as a palliative measure. Other remedies are useless. Fresh glands on ice do not produce toxic effects, and best results are in chlorotic patients when raw sheep's gland is used. Operative.—Dyspnea stridor, rapid growth, dysphagia, deformity, exophthalmic goitre, malignancy and emaciation, one or all indicate operation. Removal of all or part of the gland should be given preference to the injection of iodine, zinc, iodoform, alcohol or any other solution. Excision is more radical, safer, and requires less time for recovery. Then, too, none of the neoplasm remains to be the seat of new growth, malignant or benign. All forms of new growth of the thyroid gland should be removed. Even in cases of exophthalmic goitre it should be operated. All operative experience leads to this conclusion. Great relief has been given in exophthalmic goitre. Method.—If the disease is confined to one of two lobes without an isthmus the diseased lobe may be completely removed without much likelihood of recurrence of the growth. If an isthmus be present the other gland may become involved. It is probable that the disease being confined to one lobe may be due to the absence of the isthmus. So far as possible the presence or absence of the second lobe should be determined at the time of operation. If the second lobe cannot be found the entire diseased lobe should not be removed unless it be malignant. The probabilities are that one or more supernumerary lobes are more frequently present in persons possessing but one normal lobe, no matter where the abnormal ones may be located. Supernumerary glands are more frequent upon the left side. The presence of supernumerary lobes may account for the absence of ill effects in those persons who have been subjected to the removal of an entire right or left gland. Division of the capsule will permit of a thyroid gland being enucleated with ease and with the loss of but little blood. The rapid pulse following the removal of a thyroid gland is probably due to the rapid absorption of the thyroidine in the process of repair. The pulse will sometimes become much more rapid for forty-eight to one hundred hours, reaching at times 160 per minute, but it will at the end of this time subside to 80 or 90. If there is any pathologic tissue that should be excised it is that of the thyroid gland. There are none of the major operations in which the mortality is less.

---

### CASES IN HÆMATHERAPY FROM SOUND VIEW HOSPITAL.

BY T. J. BIGGS, M.D., STAMFORD, CONN.

### CASE I. SKIN-GRAFTING WITH CALLUS SHAVINGS, IN BLOOD.

Mary M., aged sixty years, Irish. Diagnosis, ulcer of leg. Patient admitted to hospital, March 3, 1902. She had a large varicose ulcer situated over the tibia, about 3½ by 2 inches. This condition had existed for nine years, and during that time in spite of all treatment employed had never entirely healed. It had been skin-grafted in the old way, three times unsuccessfully. At the time of entering the hospital the patient suffered so severely from pain that at times she would cry out. She was put to bed, secretions regulated, the ulcer cleaned up by means of a dermal curette, and dressed for the first twenty-four hours with a Thiersch pack. On the morning of March 5th, after the surface had been thoroughly cleaned up, a bovinine-pure pack was applied and kept wet with the bovinine for twenty-four hours.

On the morning of the 7th, I determined to employ grafts secured from a callus on the small toe, in order to demonstrate the technique of this mode of skin-grafting to five visiting physicians. The mode of procedure was as follows: The callus was thoroughly scrubbed up, and the external layers scraped off. Then thin sections of the layers next to the true skin were obtained by means of a very keen razor. Nine of these were deposited on the ulcerous surface. Over these were laid strips of perforated rubber tissue, then strips of plain bi-sterilized gauze saturated in bovinine, and a bandage applied. The nurse was instructed to keep the dressings wet with bovinine pure. This dressing was removed on the 14th, and it was found, much to the delight and astonishment of the visiting physicians, that out of the nine grafts employed eight were firmly adherent and in a healthy growing condition. The ninth had become displaced and was removed. The wound was now dressed with bovinine pure; the dressings being kept wet, and changed once in twenty-four hours. Coincident with the local dressings, from the outset, the patient had been given a wineglassful of bovinine in milk alternating with wine and beer every three hours. On March 24th she was discharged cured, the entire surface having become covered with new healthy skin.

This experiment has been employed frequently enough by me to demonstrate that where the technique is carefully followed it will in the majority of cases yield the most gratifying results. A point of interest in this case, and a usual one, is that from the day of the first dressing of the bovinine up to the time the patient was discharged, she was relieved of all pain.

CASE II. SKIN-GRAFTING WITH SKIN-SCRAPINGS, IN BLOOD.

Anna H., aged twelve years, American. Diagnosis, burn of right hand. Patient was admitted to hospital March 8, 1902. As a result of the burn she had on the back of her hand an ulcerous surface 2 by 1¾ inches, very painful, and in spite of three months' treatment had refused to heal. It was impossible in this case to secure skin-grafts, and as I wished to demonstrate to the visiting physicians who were present the efficacy of *skin scrapings* as a means of bringing about a rapid healing of small surfaces where grafts could not be obtained, with an ordinary vaccinating comb I secured skin scrapings from the little patient's arms, legs and back. These were deposited within the periphery and dressed as in the other case. The dressing was kept wet with bovinine pure until the morning of the 16th, at which time it was removed, and to the delight of the visiting physicians as before, the surface was found to be almost entirely healed, there remaining unhealed only a small space about the size of a ten-cent piece, in the center. The wound was now dressed with bovinine pure, and the nurse ordered to change it every twenty-four hours. Internally the patient had been getting a teaspoonful of bovinine every two hours in peptonized milk. March 24th she was discharged cured.

CASE III. SKIN GRAFTS HEALED IN SIX DAYS WITH BLOOD.

Arnold L., aged twenty-four years, German. Diagnosis, wound of the left cheek, the result of being thrown from a street car. Patient admitted to hospital March 10, 1902. The wound was filled with gravel and dirt, and involved almost the entire side of the face. A space in the center of the cheek, 2 by 1½ inches, was completely denuded of skin. In this case it being desirable to have the wound heal rapidly and with no evidence of scar, I determined to use grafts of normal skin sufficiently large to entirely cover the denuded surface. These grafts were secured from the patient's arms. The wound was dressed as in the other cases; the dressing being kept wet with bovinine. March 17th the dressing was removed, and the wound was entirely healed, leaving no evidence of a scar whatever; but around the periphery there was some decided redness. This is probably the most rapid case of healing of this class on record.

CASE V.—GREAT TWELVE-YEAR OLD ULCER HEALED WITH APPLIED BLOOD, WITHOUT SKIN GRAFTING.

Mike L.; age fifty-seven, Irish. Diagnosis, ulcer of left leg. Admitted to hospital March 3, 1902. This condition was of about twelve years standing, and during that time had never entirely healed. He had been treated at various hospitals and at various clinics and by private physicians, but said that he got no special relief. The ulcer was a large one situated on the calf of the leg, being 4 by 3¾

inches. It was covered with unhealthy granulations which exuded a foul-smelling purulent discharge. The surface of the ulcer was thoroughly cleaned up with a dermal curette, and dressed with a wet Thiersch pack. This was kept wet and not changed in twenty-four hours. At the end of the twenty-four hours this dressing was removed, the wound thoroughly cleansed with bovinine and hydrozone reaction, followed by Thiersch irrigation, and dressed with bovinine pure. The bovinine dressings were changed twice in twenty-four hours, and the patient got a wineglassful of bovinine internally, every three hours. March 23d, the ulcer had healed with the exception of a small space at the upper periphery. This was touched up with a twenty-five per cent. solution of pyrozone, and dressed with bovinine pure; the dressings being renewed twice in twenty-four hours. March 30th, the patient was discharged cured; the ulcer having become covered with healthy skin, and no scar tissue, it being almost impossible to tell it from the surrounding skin, the only difference being that it was a little redder.

CASE IV.—VIOLENT ENDOMETRITIS CURED BY APPLIED BLOOD, WITHOUT CURETTAGE.

Florence B., age thirty years; American. Diagnosis, endometritis. Patient admitted to hospital March 2, 1902. She was greatly anemic and emaciated. Was so weak that she had to be carried from the carriage to her bed. Discharge was so profuse that unless proper appliances were used it would run from her almost constantly.

This condition had existed for four years, and during that period she had been twice curetted, but no result or relief obtained. Examination revealed the uterus to be in a highly diseased condition. So much so that I advocated a vaginal hysterectomy, or at least a thorough curettment. To these propositions both the patient and her friends absolutely declined to agree, and begged that I employ some other treatment. I therefore, without any promise of result, determined to employ the bovinine injections and applications. On the 3d of March after the patient's secretions had been regulated I commenced treatment by washing out the uterus and injecting a solution of bovinine and salt water, two-thirds bovinine and one-third salt water, and tamponing the vagina with bovinine pure. Internally she was given two teaspoonfuls of bovinine every hour in peptonized milk and a little water. The vaginal injections and tamponing were employed twice in twenty-four hours up to March 14th. At this time the discharge had entirely ceased and the uterus was becoming smaller. The uterine washings were now employed once in twenty-four hours and, instead of bovinine tamponings, vaginal injections of the bovinine pure. Internally the bovinine was increased to a wineglassful every two hours. March 18th, the patient was up, and went for a short walk, and returned in splendid condition. Had gained four and three-fourths pounds in weight. On March 23d, the uterine injections were discontinued, and the vaginal injections employed once in twenty-four hours. At this time the uterus had assumed its normal size, and all evidence of inflammation had disappeared. The patient was looking and feeling splendidly. Theretore local treatment was discontinued. April 1st, she was discharged cured, but instructed to return at intervals for examination and continue the bovinine internally indefinitely.

This case was certainly an extreme one and by all gynecologists an operation would have been deemed, I think, an absolute necessity.

